# The Complete Chloroplast Genome Sequences of *Aconitum pseudolaeve* and *Aconitum longecassidatum*, and Development of Molecular Markers for Distinguishing Species in the *Aconitum* Subgenus *Lycoctonum*

**DOI:** 10.3390/molecules22112012

**Published:** 2017-11-21

**Authors:** Inkyu Park, Sungyu Yang, Goya Choi, Wook Jin Kim, Byeong Cheol Moon

**Affiliations:** K-herb Research Center, Korea Institute of Oriental Medicine, Daejeon 305-811, Korea; pik6885@kiom.re.kr (I.P.); sgyang81@kiom.re.kr (S.Y.); serparas@kiom.re.kr (G.C.); ukgene@kiom.re.kr (W.J.K.)

**Keywords:** *Lycoctonum*, plastid, Ranunculaceae, medicinal plant, species identification

## Abstract

*Aconitum pseudolaeve* Nakai and *Aconitum longecassidatum* Nakai, which belong to the *Aconitum* subgenus *Lycoctonum*, are distributed in East Asia and Korea. *Aconitum* species are used in herbal medicine and contain highly toxic components, including aconitine. *A*. *pseudolaeve,* an endemic species of Korea, is a commercially valuable material that has been used in the manufacture of cosmetics and perfumes. Although *Aconitum* species are important plant resources, they have not been extensively studied, and genomic information is limited. Within the subgenus *Lycoctonum,* which includes *A*. *pseudolaeve* and *A*. *longecassidatum*, a complete chloroplast (CP) genome is available for only one species, *Aconitum barbatum* Patrin ex Pers. Therefore, we sequenced the complete CP genomes of two *Aconitum* species, *A. pseudolaeve* and *A. longecassidatum*, which are 155,628 and 155,524 bp in length, respectively. Both genomes have a quadripartite structure consisting of a pair of inverted repeated regions (51,854 and 52,108 bp, respectively) separated by large single-copy (86,683 and 86,466 bp) and small single-copy (17,091 and 16,950 bp) regions similar to those in other *Aconitum* CP genomes. Both CP genomes consist of 112 unique genes, 78 protein-coding genes, 4 ribosomal RNA (rRNA) genes, and 30 transfer RNA (tRNA) genes. We identified 268 and 277 simple sequence repeats (SSRs) in *A. pseudolaeve* and *A. longecassidatum*, respectively. We also identified potential 36 species-specific SSRs, 53 indels, and 62 single-nucleotide polymorphisms (SNPs) between the two CP genomes. Furthermore, a comparison of the three *Aconitum* CP genomes from the subgenus *Lycoctonum* revealed highly divergent regions, including *trnK*-*trnQ, ycf1*-*ndhF*, and *ycf4-cemA.* Based on this finding, we developed indel markers using indel sequences in *trnK*-*trnQ* and *ycf1*-*ndhF*. *A. pseudolaeve, A. longecassidatum*, and *A. barbatum* could be clearly distinguished using the novel indel markers AcoTT (*Aconitum trnK*-*trnQ*) and AcoYN (*Aconitum ycf1*-*ndhF*)*.* These two new complete CP genomes provide useful genomic information for species identification and evolutionary studies of the *Aconitum* subgenus *Lycoctonum*.

## 1. Introduction

Chloroplasts (CPs) play important functional roles in photosynthesis, biosynthesis, and metabolism of starch and fatty acids throughout the plant life cycle [1]. The angiosperm CP genome is a circular molecule with a quadripartite structure consisting of large single-copy (LSC) and small single-copy (SSC) regions and two copies of an inverted repeat (IR) region. Typically, the CP genomes of higher plants contain 110–120 genes, encoding proteins, transfer RNAs (tRNAs), and ribosomal RNAs (rRNAs), and are 120–160 kb in length. The structure, gene content, and gene orientation is highly conserved at the genus level [2]. Even within genera, however, CP genomes have undergone size changes, structure rearrangement, contraction and expansion of IRs, and even pseudogenization due to adaptations to their host plants’ environments [1,3]. Since the first CP genome was reported (for tobacco), more than 9000 complete CP genomes (October 2017) have been deposited in GenBank [4]. These data contain valuable information that has been used in reconstruction of high-resolution phylogenic trees, development of markers for species identification, and for evolutionary genetic studies [3,5,6,7,8]. Universal DNA barcoding is useful tool for species identification. However, in the *Aconitum* species, it is difficult to distinguish using universal DNA barcode. The complete CP genome is useful tool to solve DNA barcode limitation.

Although the CP genome is more highly conserved than the nuclear genome, it frequently contains insertions/deletions (indels) and single-nucleotide polymorphisms (SNPs) [9,10]. These variations have been used to estimate divergence times among evolutionarily-related species [11,12,13]. Also, these mutations can be used as markers to rapidly distinguish species [14,15,16]. Several studies report the use of indels and SNP mutations from complete CP genomes for species identification in *Panax ginseng*, buckwheat, *Aconitum*, and other genera [7,14,15]. Specifically, the CP genomes of nine *Panax ginseng* cultivars were sequenced, and six markers consisting of three indels and three derived cleaved amplified polymorphic sequences (dCAPS) were developed to distinguish subspecies of *P. ginseng* through comparative analysis [14]. Three divergent coding regions (*rpoC1*, *cpoC2* and *ycf1*) and three intergenic sequence (IGS) regions (*rpl32-trnL*, *rpl16-trnQ* and *trnQ-trnT*) were used for this purpose. Tartary buckwheat (*Fagopyrum tataricum*) and common buckwheat (*Fagopyrum esculentum*) were classified based on six indel markers from one coding region, *ycf1*, and four IGS regions (*trnS-trnG*, *rpoB-trnC*, *psbM-trnD*, and *ndhC-trnV*) [15]. *Aconitum coreanum* (H.Lév.) Rapaics also has a unique insertion mutation (in the *ndhC*-*trnV* region) not present in other *Aconitum* species, and this region was used to develop a sequence characterized amplified region marker specific for *A. coreanum* [7]. Thus, indel and SNP mutations provide useful information for identification of species, phylogenic tree analysis, and population studies. Such markers can also overcome the limits of universal DNA barcodes.

*Aconitum* species are largely classified into three subgenera, *Aconitum*, *Lycoctonum*, and *Gymnaconitum*, which together comprise approximately 400 species [17]. These plants, which belong to the family Ranunculaceae, are distributed in the Northern hemisphere. In particular, *Aconitum pseudolaeve* Nakai and *Aconitum longecassidatum* Nakai are widely distributed in East Asia [17], and *A. pseudolaeve* is a valuable plant endemic to Korea [18]. *A. pseudolaeve* and *A. longecassidatum* are 30–80 cm in height, with mean stem length of 65 cm, short branches, and retrorse yellowish pubescent and pentagonal-reniform leaf blades. Although these two species are very similar morphologically, their inflorescences differ slightly. First, the bracteoles of *A. pseudolaeve* are 2–3 times longer than the pedicel, whereas those of *A. longecassidatum* are as long as or slightly shorter than the pedicel. Second, the pistils of *A. pseudolaeve* have recurved hair. However, *A. longecassidatum* forms glabrous pistils [19].

Notwithstanding these morphological distinctions, the two species are used indiscriminately as herbal medicine as Radix Lycoctoni [20]. The roots of both plants are used to relieve neuralgia, reduce fever, and lower blood pressure [20], and extracts from *A. pseudolaeve* are purported to have anti-aging and anti-diabetes effects [21]. For these reasons, the extract of *A. pseudolaeve* is used in cosmetic compounds, as well as in herbal medicine [22]. In particular, perfume based on *A. pseudolaeve* is used in aromatherapy intended to improve emotional stability [23]. Although the two *Aconitum* species have medicinal and commercial value, they are often used without species identification. The ability to distinguish the two species would improve the medicinal potential, quality control, and stability of commercial products containing material from these plants.

In this study, we sequenced the complete CP genomes of *A. pseudolaeve* and *A. longecassidatum*. Comparative analysis of the two CP genomes revealed highly divergent regions and potential indels, simple sequence repeats (SSRs), and SNPs. In addition, based on comparative CP genome analysis of *A. pseudolaeve*, *A. longecassidatum*, and *A. barbatum* Patrin ex Pers., we developed indel markers to distinguish three species of the subgenus *Lycoctonum* based on divergent regions of the CP genome. These results will provide useful genetic tools for identification of *Aconitum* species of the subgenus *Lycoctonum*, and also will inform genomic resources for evolutionary studies of these plants.

## 2. Results and Discussion

### 2.1. CP Genome Organization of A. pseudolaeve and A. longecassidatum

**Figure 1:**
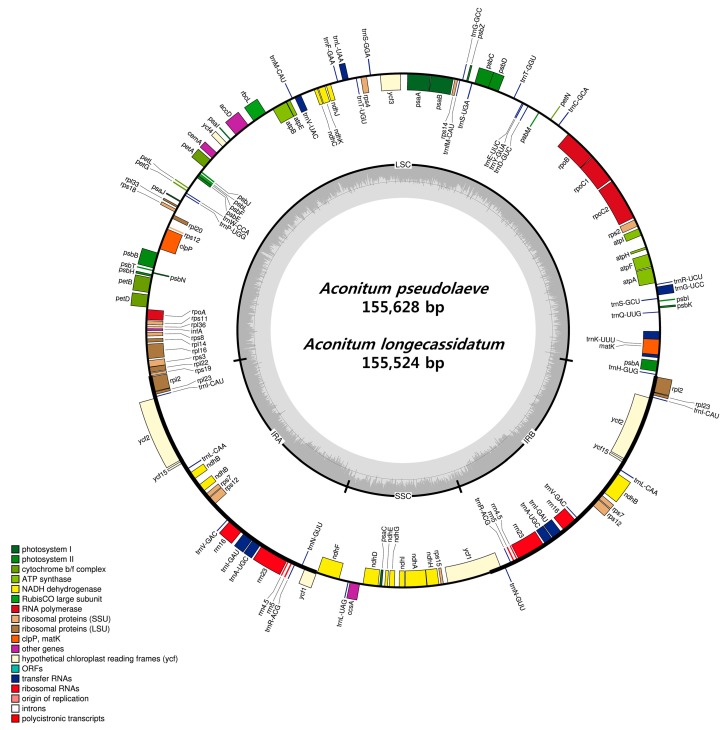
Circular gene map of *A. pseudolaeve* and *A. longecassidatum*. Genes drawn inside the circle are transcribed clockwise, and those outside the circle counterclockwise. The darker gray in the inner circle represents (GC) content. The gene map corresponds to *A. pseudolaeve*. LSC: large single copy; IR: inverted repeat; SSC: small single copy; GC: guanine-cytosine; ORF: open reading frame.

We obtained trimmed reads (approximately 2.7 Gb) from *A. pseudolaeve* Nakai and *A. longecassidatum* Nakai using the MiSeq platform. Seven and six initial CP contigs of *A. pseudolaeve* and *A. longecassidatum*, respectively, were de novo assembled from low-coverage whole-genome sequence. The complete CP genomes of *A. pseudolaeve* and *A. longecassidatum* are 155,628 and 155,524 bp in length, with approximately 345× and 222× coverage, respectively (Table S1). The complete CP genomes of both *Aconitum* species have the quadripartite structure characteristic of most angiosperms (Figure 1): a pair of IRs (51,854 bp and 52,108 bp in *A. pseudolaeve* and *A. longecassidatum*, respectively) and two single-copy regions (LSC, 86,683 bp and 86,466 bp; SSC, 17,091 and 16,950 bp in *A. pseudolaeve* and *A. longecassidatum*, respectively) (Figure 1 and Table 1). The guanine-cytosine (GC) contents of the two *Aconitum* CP genomes are 38.0% and 38.1%, with IR regions having higher GC content (43.1% and 43.0% in *A. pseudolaeve* and *A. longecassidatum*, respectively) than LSC (36.1% in both species) and SSC regions (32.6% and 32.7% in *A. pseudolaeve* and *A. longecassidatum*, respectively) (Table S2). Thus, the *A. pseudolaeve* and *A. longecassidatum* CP genomes are AT-rich, similar to those of other land plants [7,24,25].

**Figure 2:**
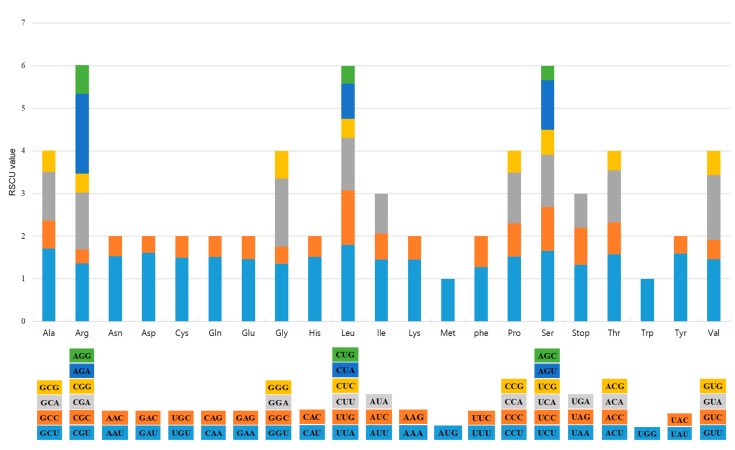
Codon content for the 20 amino acids and stop codon in 78 protein-coding genes in the *A. pseudolaeve* Nakai and *A. longecassidatum* Nakai CP genomes. Colors in the column graph reflect codons in the same colors shown in the lower part of the figure. RSCU: relative synonymous codon usage; Ala: alanine; Arg: arginine; Asn: asparagine; Asp: asparagine; Cys: cysteine; Gln: glutamine; Glu: glutamic acid; Gly: glycine; His: histidine; Leu: leucine; Ile: isoleucine; Lys: lysine; Met: methionine; Phe: phenylalanine; Pro: proline; Ser: serine; Thr: threonine; Trp: tryptophan; Tyr: tyrosine; Val: valine.

Gene content and gene order were similar to those in other *Aconitum* CP genomes [7]. Both the *A. pseudolaeve* and *A. longecassidatum* CP genomes have 112 unique genes, including 78 protein-coding genes, 30 tRNAs, and 4 rRNAs (Table 2). Of these, 18 genes are present as duplicates: seven tRNAs, four rRNAs, and seven protein-coding genes (*ndhB*, *rpl2*, *rpl23*, *rps7*, *rps12*, *ycf1* and *ycf2*). The two *Aconitum* CP genomes each have 17 intron-containing genes, including 15 genes with a single intron and two with two introns; *rps12* is trans-spliced, as in other *Aconitum* species. The *trnK-UUU* gene (2526 bp in *A. pseudolaeve* and 2525 bp in *A. longecassidatum*) has the longest intron region with *matK* (Table 3). The *ndhD* and *rpl2* genes use the alternative start codon ACG; *rps19* and *ycf2* use GTG; and *rpl2* and *rps19* use ACG or GTG, as previously reported [26]. *rps16* contains one exon deletion in both *A. pseudolaeve* and *A. longecassidatum*. *ycf1* is present in two copies; one *ycf1* copy was located in the boundary region between IRa and SSC in *A. pseudolaeve*. The 78 protein-coding sequences comprise 26,459 codons in *A. pseudolaeve* and 26,487 codons in *A. longecassidatum* (Table S3). Leucine and isoleucine are abundant in both CP genomes. (Figure S1). Two or more synonymous codons are used for all amino acids except methionine and tryptophan (Figure 2). The most preferred synonymous codons (relative synonymous codon usage; RSCU > 1) contain A or T in the third position, contributing to the AT bias, as in other *Aconitum* CP genomes [7,27]. Arginine, leucine, and serine are each represented by six synonymous codons with higher RSCU values [26,28]. This may be for protecting protein mutations due to important amino acids in biosynthesis. The sequence of both *Aconitum* CP genomes consists of 58% protein-coding genes, 1.8% tRNA genes, and 5.8% rRNA; the remaining 41.4% is comprised of non-coding regions, including pseudogenes and introns. *ycf1* and *rps16* are pseudogenes, as in other *Aconitum* species [27,29]. Both CP genomes are very similar in terms of gene order, content, and structure, and are highly conserved relative to the CP genomes of other *Aconitum* species [7,27,29].

### 2.2. Repeat Analysis in Two Aconitum Chloroplast Genomes

**Figure 3:**
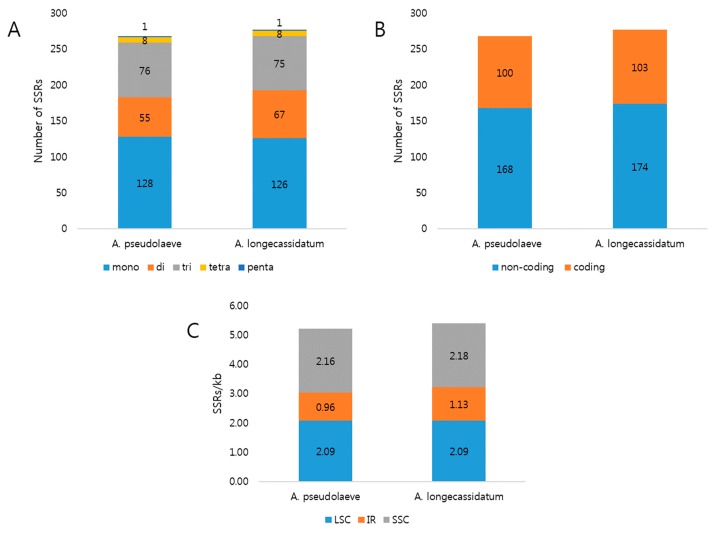
Distribution of simple sequence repeats (SSRs) in *A. pseudolaeve* Nakai and *A. longecassidatum* Nakai CP genomes. (**A**) Distribution of SSR types in the two *Aconitum* CP genomes. (**B**) Distribution of SSRs between coding and non-coding regions. (**C**) Number of SSRs per unit length in the indicated genomic regions of *Aconitum* CP genomes. CP: chloroplast; LSC: large single copy; IR: inverted repeat; SSC: small single copy.

Microsatellites or simple sequence repeats (SSRs) are made up of abundant tandem repeat sequences consisting of 1–6-nt motifs. These elements are useful markers due to their high degree of polymorphism. In addition, SSRs are used for phylogenic analysis in population genetics [30,31]. We identified SSR loci, revealing 268 and 277 SSRs in the CP genomes of *A. pseudolaeve* Nakai and *A. longecassidatum* Nakai, respectively (Figure 3). Mononucleotides were the most abundant motifs, constituting 128 (47.8%) and 126 (45.5%) of the SSRs in *A. pseudolaeve* and *A. longecassidatum*, respectively. Approximately 37% of SSRs were distributed in coding regions. More SSRs were present in single-copy regions than in IR regions. To detect potential SSR loci for development of markers to distinguish the two *Aconitum* species, we identified 36 SSR indels consisting of A or T motifs, ranging in length from 1 to 6 bp (Table 4). The region exhibiting the greatest difference between species was a 6-bp SSR in *psbM-trnD* (IGS). One SSR is present in an exon of *ndhG* (Table 4).

Repeat sequences play important evolutionary roles, influencing changes in genome structure such as duplication and rearrangement [32]. We detected tandem repeats of 20 or 21 bp in *A. pseudolaeve* and 19 bp in *A. longecassidatum* (Table S4). Most tandem repeats were located in IGS regions, and were present in both the *ycf1* and *ycf2* genes. Fourteen repeats were shared between the two *Aconitum* species. Three tandem repeats were located in *ycf2*, and two in *trnK-rps16*. In both species, six palindromic repeats were present, ranging in size from 21 to 33 bp (Table 5). In particular, the *ycf2* gene contained short tandem repeats as well as a palindromic repeat.

### 2.3. Comparison of the Chloroplast Genomes of A. pseudolaeve Nakai, A. longecassidatum Nakai and Aconitum barbatum Patrin ex Pers.

Based on a phylogenetic analysis of the CP genome sequence, *A. pseudolaeve* and *A. longecassidatum* have been clustered within the *Aconitum* subgenus *Lycoctonum*, genetically closest to *A. barbatum* [27]. Consistent with this, the CP genomes of *A. pseudolaeve* and *A. longecassidatum* are 99.7% similar, with nearly identical genome structure, gene content, and gene order, although the single-copy (LSC and SSC) and IR regions differ slightly. The LSC and IR regions of the *A. barbatum* CP genome are slightly longer than those of *A. pseudolaeve* and *A. longecassidatum*, whereas the SSC regions are shorter. Thus, overall, the CP genomes of the three *Aconitum* species are very similar.

**Figure 4:**
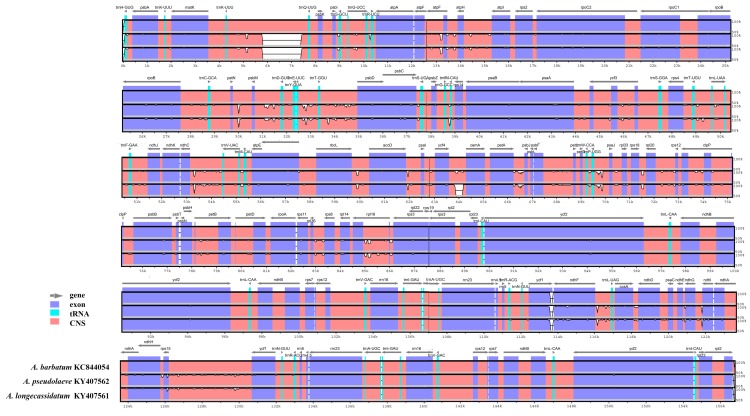
Comparison of *A. pseudolaeve* Nakai, *A. longecassidatum* Nakai and *Aconitum barbatum* Patrin ex Pers. chloroplast (CP) genomes using mVISTA. Complete CP genomes of *A. pseudolaeve* and *A. longecassidatum* were compared with *A. barbatum* as a reference. Blue block: conserved gene; sky-blue block: tRNA and rRNA; red block: intergenic region. White peaks are sequence variation regions between *A. barbatum* and *A. pseudolaeve,* and *A. barbatum* and *A. longecassidatum*. CP: chloroplast; tRNA; transfer RNA; CNS: conserved non-coding sequence.

**Figure 5:**
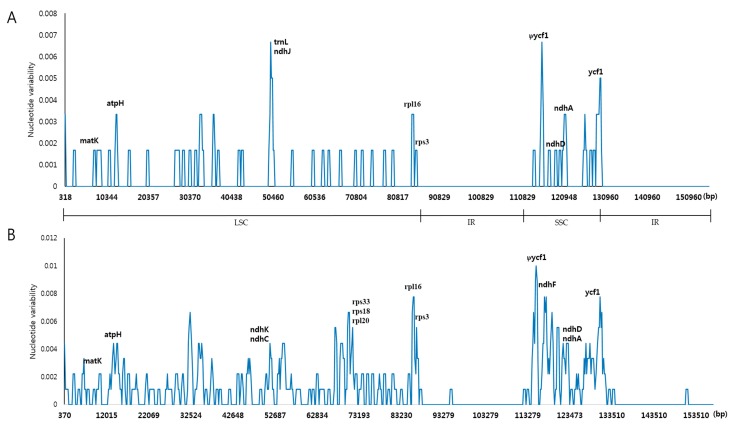
Sliding-window analysis of CP genomes. (**A**) Comparison of the nucleotide variability (Pi) between *A. pseudolaeve* Nakai and *A. longecassidatum* Nakai CP genomes; (**B**) Comparison of the nucleotide variability (Pi) among *Aconitum* subgenus *Lycoctonum* CP genomes, including *A. barbatum* Patrin ex Pers., *A. pseudolaeve,* and *A. longecassidatum.* CP: chloroplast

To identify divergent regions among the three species, we performed sequence alignment against the *A. barbatum* CP genome (Figure 4). The greatest divergence was observed in non-coding regions. In particular, *A. barbatum* contains a large insertion in *trnK-trnQ* that is not present in the other two species. Smaller divergent regions are present in *petN-psbM*, *trnT-trnL*, *ndhC-trnV*, *rbcL-accD*, and other loci; *A. barbatum* has more divergent regions than the other two species. Almost all divergent regions are located in non-coding regions such as *trnR-atpA*, *trnT-psbD*, *ycf4-cemA*, *ndhC-trnV*, and *ycf1-ndhF*. As noted above, coding regions are highly conserved between *A. pseudolaeve* and *A. longecassidatum* (Figure S2). The most divergent regions were found in non-coding regions such as *trnR-atpA*, *trnT-psbD*, *ycf4-cemA*, *ndhC-trnV*, and *ycf1-ndhF* (Figure S2). In addition, to analyze divergence at the sequence level among the three *Aconitum* CP genomes, we also calculated the nucleotide variability (Pi) value (Figure 5). As expected, IR regions are dramatically conserved among the three species. In other words, single-copy (LSC and SSC) regions are more variable than IR regions. The divergence among the three *Aconitum* species is greater than that between *A. pseudolaeve* and *A. longecassidatum*. As shown in Figure 5, a few regions exhibited divergence (*atpH*, *trnL*, *ndhJ*, *rpl16*, *ycf1*, and *ndhA*), with a maximal Pi value of 0.7%.

### 2.4. Indel and SNP Mutation between A. pseudolaeve and A. longecassidatum

Indels and SNPs are common events in the evolution of higher plant CP genomes [9,33,34,35]. These mutations provide information that is useful for resolving evolutionary relationships in phylogenetic analyses of related taxa [36]. We detected 61 indels between *A. pseudolaeve* Nakai and *A. longecassidatum* Nakai (Table S5), of which 53 are located in IGS regions and the remaining eight are in coding regions. Most indels range from 1 to 6 bp, and eight indels are longer than 10 bp; the longest indel, in ycf4-cemA, has a length of 256 bp. No indels were found in IR regions. Comparison of the *Aconitum* species revealed a large insertion (1582 bp) in *A. barbatum* Patrin ex Pers. not present in *A. pseudolaeve* or *A. longecassidatum*. *trnK*-*trnQ* is highly conserved between *A. pseudolaeve* and *A. longecassidatum*.

**Figure 6:**
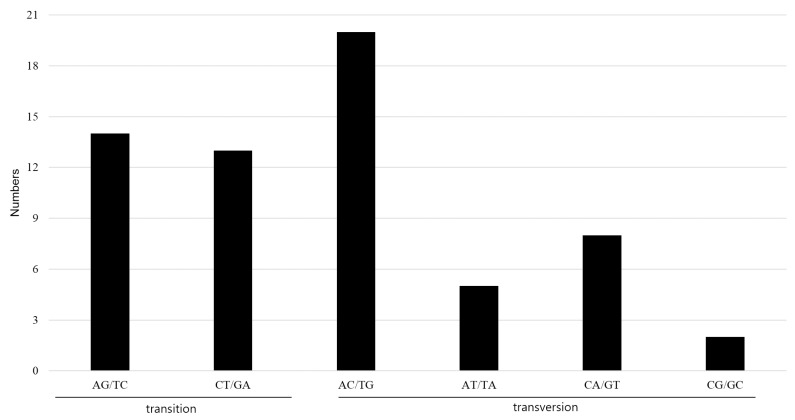
The nucleotide substitution pattern in *A. pseudolaeve* Nakai and *A. longecassidatum* Nakai CP genomes. The patterns were divided into six types, as indicated by the six non-strand-specific base-substitution types (i.e., numbers of considered G to A and C to T site sites for each respective set of associated mutation types). The *A. pseudolaeve* chloroplast genome was used as a reference. CP: chloroplast

We also detected 62 SNPs consisting of 27 transitions (Ts) and 35 transversions (Tv) between two CP genomes (Figure 6 and Table S6). The ratio of Ts/Tv was 1:0.77, similar to that of other CP genomes [9,37]. Some nucleotides were substituted A-to-C and T-to-G (32%). Substitution of C-to-G and G-to-C showed the lowest frequency (3%). Of these 62 SNPs, 26 are located in coding regions. In particular, the *ycf1* gene contains nine SNPs (three Ts, six Tv), and thus represents a hotspot region containing clustered variation [9,38]. We detected no non-synonymous SNPs between *A. pseudolaeve* and *A. longecassidatum*.

### 2.5. Development and Validation of the Indel Marker for Authentication of Three Species in the Aconitum Subgenus Lycoctonum

**Figure 7:**
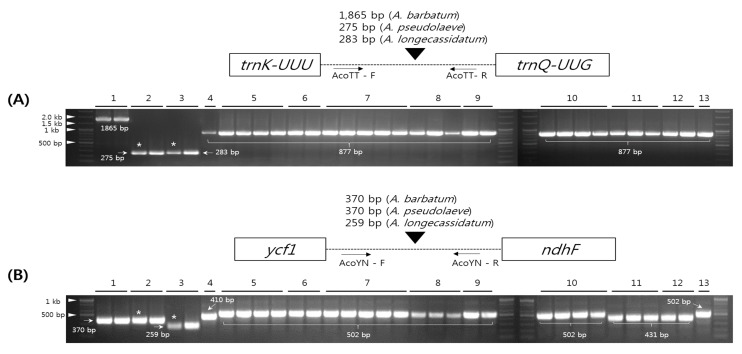
Schematic diagram of development of indel markers (AcoTT and AcoYN) in *A. pseudolaeve* Nakai, *A. longecassidatum* Nakai, and *A. barbatum* Patrin ex Pers. (**A**) Primers for AcoTT were tested in 13 *Aconitum* species. AcoTT length of *A. barbatum* was measured in the CP sequence; (**B**) Primers for AcoYN were validated in 13 *Aconitum* species: (1) *A. barbatum*, (2) *A. pseudolaeve*, (3) *A. longecassidatum*, (4) *A. coreanum* (H.Lév.) Rapaics, (5) *A. carmichaelii* Debeaux, (6) *A. voluvile* var. *pubescens* Regel, (7) *A. jaluense* var. *triphyllum* (Nakai) U.C.La, (8) *A*. *kusnezoffii* Rchb., (9) *A*. *jaluense* Kom., (10) *A*. *austrokoreense* Koidz., (11) *A*. *monanthum* Nakai, (12) *A*. *kirinense* Nakai, (13) *A*. *chiisanense* Nakai. * The CP genomes of *A. pseudolaeve* and *A. longecassidatum* were completed in this study. AcoYN: *Aconitum ycf1-ndhF*; AcoTT: *Aconitum trnK-trnQ*. CP: chloroplast.

Indel regions are commonly used for development of markers because they are easy to detect, and it is straightforward to design suitable primers for them [14,15,39]. We developed indel markers using the sequence variability of the large indel regions in *A. pseudolaeve* Nakai, *A. longecassidatum* Nakai, and *A. barbatum* Patrin ex Pers. (Figure 4). Specifically, we designed indel primers based on the conserved regions of *trnK-trnQ* and *ycf1-ndhF*. AcoTT (*Aconitum trnK*-*trnQ*) and AcoYN (*Aconitum ycf1*-*ndhF*) primers successfully amplified the predicted products in all three *Aconitum* species (Figure 7 and Data S1). *A. pseudolaeve* and *A. longecassidatum* exhibit a small length difference in AcoTT, whereas *A. barbatum* exhibits a longer PCR product than the other two species, as expected. As shown in Figure 7, *A. barbatum*, *A. pseudolaeve*, and *A. longecassidatum* yielded amplicons of 1865 bp, 275 bp, and 283 bp, respectively. Furthermore, *A. longecassidatum* has a 6-bp insertion relative to *A. pseudolaeve*. In AcoYN, only *A. longecassidatum* (259 bp) exhibits a difference to *A. pseudolaeve* and *A. barbatum* (370 bp). In the previous study analyzing molecular phylogeny based on the CP genome sequences of *Aconitum* species, we found that two *Aconitum* subgenera, *Aconitum* and *Lycoctonum*, were clearly classified [7]. To confirm the variability of indel regions between *Aconitum* species and subgenera, we conducted analysis of PCR amplification profiles using the indel markers AcoTT and AcoYN, and a total 27 samples of *Aconitum* species (nine species and one variety) consisting of *Aconitum* subgenera *Aconitum* and *Lycoctonum* (Figure 7). Interestingly, all 27 other *Aconitum* samples yielded only the 877-bp amplicon for AcoTT, but three band patterns for AcoYN (Figure 7): the PCR products for *A. monanthum* Nakai and *A. kirinense* Nakai were 431 bp; that of *A. coreanum* was 410 bp; and those of the other species were 502 bp. However, *A. longecassidatum* was clearly distinguished from the other *Aconitum* species. Taken together, these findings confirm that the three *Aconitum* species each have specific sequences, and that it is possible to distinguish them from other *Aconitum* species. 

Because *A. pseudolaeve* and *A. longecassidatum* have highly conserved CP genome structures, it is difficult to develop markers for the *Aconitum* genus that can distinguish at the species level. Furthermore, *A. pseudolaeve* and *A. longecassidatum* had consistent sequences in the universal DNA barcode regions such as internal transcribed spacer (ITS), *matK*. By comparative analysis, however, we detected genetic variants and used them to develop indel markers. Specifically, the *trnK-trnQ* region could distinguish *A. pseudolaeve*, *A. longecassidatum*, and *A. barbatum*. These three species of the *Aconitum* subgenus *Lycoctonum* contain specific indel regions not present in the subgenus *Aconitum*. In this study, we overcame the limitations of universal DNA barcodes for inter-species identification. Thus, our indel markers (AcoTT and AcoYN) will be useful in identification of *A. pseudolaeve*, *A. longecassidatum*, and *A. barbatum* (Table 6). Furthermore, we confirmed that these markers can be used to distinguish *Aconitum* at the subgenus level. It is likely that the subgenus *Aconitum* exhibits greater conservation (i.e., less variation) than the subgenus *Lycoctonum*. Although only a few *Aconitum* species were used in this study, our findings will contribute to species classification in *Aconitum* subgenus *Lycoctonum*.

## 3. Materials and Methods

### 3.1. Plant Materials and Genome Sequencing

We collected fresh leaves of *A. pseudolaeve* Nakai (KIOM201401010986) and *A. longecassidatum* Nakai (KIOM201401010506) from medicinal plantations in Korea, and subjected the samples to CP genome sequencing. *A. pseudolaeve* and *A. longecassidatum* were given identification numbers, and specimens were registered in the Korean Herbarium of Standard Herbal Resources (Index-Herbarium code KIOM) at the Korea Institute of Oriental Medicine (KIOM) [20]. DNA was extracted using the DNeasy Plant Maxi kit (Qiagen, Valencia, CA, USA). Illumina paired-end sequencing libraries were constructed and generated using MiSeq platform (Illumina, San Diego, Valencia, CA, USA).

### 3.2. Assembly and Annotation of Two Aconitum Species

CP genomes were obtained by de novo assembly from low-coverage whole-genome sequence data. Trimmed paired-end reads (Phred scores ≥ 20) were assembled using CLC Genome Assembler (ver. 4.06 beta, CLC Inc, Aarhus, Denmark) with default parameters. The principal contigs representing the CP genome were retrieved from total contigs using Nucmer [40] using the CP genome sequence of *Aconitum barbatum* var. *puberulum* (KC844054) as the reference sequence. Gene annotation was performed using DOGMA [41] and manual curation using BLAST. The circular maps of *A. pseudolaeve* and *A. longecassidatum* were obtained using OGDRAW [42]. Codon usage and base composition analysis of CP genomes were performed using MEGA6 [43]. NCBI accession numbers of CP genome sequences are KY407562 and KY407561 for *A. pseudolaeve* and *A. longecassidatum*, respectively.

### 3.3. SSR, Tandem, and Palindromic Repeat Analysis in Two Aconitum CP Genomes

Tandem repeats were ≥20 bp with minimum alignment score and maximum period size set at 50 and 500, respectively, and identity of repeats was set at ≥90% [44]. SSRs were detected using MISA [45] with the minimum repeat numbers set to 10, 5, 4, 3, 3 and 3 for mono-, di- tri- tetra-, penta-, and hexanucleotides, respectively. IRs were detected using the Inverted Repeats Finder [46] with default parameters. IRs were required to be ≥20 bp in length with 90% similarity. 

### 3.4. Comparative Analysis of CP Genomes of A. pseudolaeve and A. longecassidatum

The mVISTA program [47] was used to compare the CP genomes of *Aconitum barbatum* var. *puberulum* (KC844054), *A. pseudolaeve,* and *A. longecassidatum*. To calculate nucleotide variability (Pi) between CP genomes, we performed sliding-window analysis using DnaSP version 5.1 [48] with a window length of 600 bp and step size of 200 bp. Indels and SNPs were analyzed based on sequence alignments using MAFFT [49].

### 3.5. Development and Validation of Indel Markers (AcoTT and AcoYN) Among Aconitum Species

We selected indel regions based on mVISTA similarities and designed primers using Primer-BLAST (NCBI). Indel regions were amplified from 20 ng of genomic DNA in a 20-µL PCR mixture (Solg^TM^ 2X Taq PCR smart mix 1, Solgent, Daegeon, Korea) with 10 pmol of each primer (Bioneer, Daejeon, Korea). Amplification was performed on a Pro Flex PCR system (Applied Biosystems, Waltham, MA, USA) according to the following program: (1) AcoTT primer: initial denaturation at 95 °C for 2 min; 35 cycles at 95 °C for 1 min, 61 °C for 1 min, and 72 °C for 1.5 min; and final extension at 72 °C for 5 min; and (2) AcoYN primer: initial denaturation at 95 °C for 2 min; 35 cycles at 95 °C for 50 s, 60 °C for 50 s, and 72 °C for 50 s; and final extension at 72 °C for 5 min. PCR products were separated on 2% agarose gels at 150 V for 40 min. To validate the specificity of indel markers and confirm the variability of indel regions between *Aconitum* species and subgenera *Aconitum* and *Lycoctonum*, we checked PCR amplification profiles using 27 additional samples from nine species and one variety of *Aconitum* consisting of both *Aconitum* subgenus *Aconitum* and *Lycoctonum*, which were provided from the KIOM herbarium. In addition, to confirm that the sizes of the PCR products were accurate, two samples per species were sequenced. Each PCR product was rescued from the agarose gel, subcloned into the pGEM-T Easy vector (Promega, Madison, WI, USA), and sequenced on a DNA sequence analyzer (ABI 3730, Applied Biosystems Inc., Foster City, CA, USA) to estimate sizes and verify the sequences of amplicons. 

## Figures and Tables

**Table 1:** Size comparison of two *Aconitum* species’ CP genomic regions.

Species	*Aconitum pseudolaeve*	*Aconitum longecassidatum*
Total CP genome size (bp)	155,628	155,524
LSC region (bp)	86,683	86,466
IR region (bp)	51,854	52,108
SSC region (bp)	17,091	16,950
GC content (%)	38.0	38.1
LSC (%)	36.1	36.1
IR (%)	43.1	43.0
SSC (%)	32.6	32.7
Number of genes	112	112
Protein-coding genes	78	78
rRNAs	4	4
tRNAs	30	30

CP: chloroplast; LSC: large single copy; IR: inverted repeat; SSC: small single copy; tRNAs: transfer RNAs; rRNAs: ribosomal RNAs. GC: guanine-cytosine.

**Table 2:** Genes present in the CP genomes of *A. pseudolaeve* Nakai and *A. longecassidatum* Nakai.

Gene Products of *Aconitum* Species	
Photosystem I	*psaA*, *B*, *C*, *I*, *J*
Photosystem II	*psbA*, *B*, *C*, *D*, *E*, *F*, *H*, *I*, *J*, *K*, *L*, *M*, *N*, *T*, *Z*
Cytochrome b6_f	*petA*, *B* ^(1)^, *D* ^(1)^, *G*, *L*, *N*
ATP synthase	*atpA*, *B*, *E*, *F* ^(1)^, *H*, *I*
Rubisco	*rbcL*
NADH oxidoreductase	*ndhA* ^(1)^, *B* ^(1),(3)^, *C*, *D*, *E*, *F*, *G*, *H*, *I*, *J*, *K*
Large subunit ribosomal proteins	*rpl2* ^(1),(3)^, *14*, *16* ^(1)^, *20*, *22*, *23* ^(3)^, *32*, *33*, *36*
Small subunit ribosomal proteins	*rps2*, *3*, *4*, *7* ^(3)^, *8*, *11*, *12* ^(2)–(4)^, *14*, *15*, *18*, *19*
RNA polymerase	*rpoA*, *B*, *C1* ^(1)^, *C2*
Unknown function protein-coding gene	*ycf1* ^(3)^, *2* ^(3)^, *3* ^(2)^, *4*
Other genes	*accD*, *ccsA*, *cemA*, *clpP ^(2)^*, *infA*, *matK*
Ribosomal RNAs	*rrn16* ^(3)^, *rrn23* ^(3)^, *rrn4.5* ^(3)^, *rrn5* ^(3)^
Transfer RNAs	*trnA-UGC* ^(1),(3)^, *trnC-GCA*, *trnD-GUC*, *trnE-UUC*, *trnF-GAA*, *trnG-UCC* ^(1)^, *trnG-GCC*, *trnH-GUG*, *trnI-CAU* ^(3)^, *trnI-GAU* ^(1),(3)^ *trnK-UUU* ^(1)^, *trnL-UAA* ^(1)^, *trnL-UAG*, *trnL-CAA* ^(3)^, *trnM-CAU*, *trnfM-CAU*, *trnN-GUU* ^(3)^, *trnP-UGG*, *trnQ-UUG*, *trnR-ACG* ^(3)^, *trnR-UCU*, *trnS-GCU*, *trnS-GGA*, *trnS-UGA*, *trnT-GGU*, *trnT-UGU*, *trnV-UAC* ^(1)^, *trnV-GAC* ^(3)^, *trnW-CCA*, *trnY-GUA*

^(1)^ Gene containing a single intron; ^(2)^ gene containing two introns; ^(3)^ two gene copies in inverted repeats; ^(4)^ trans-spliced gene. CP: chloroplast.

**Table 3:** Genes with introns in the *A. pseudolaeve* Nakai CP genome, and lengths of exons and introns.

Gene	Region	Exon I	Intron I	Exon II	Intron II	Exon III
*trnk-UUU*	LSC	37	2526 (2525)	35		
*trnG-UCC*	LSC	23	744 (747)	48		
*atpF*	LSC	145	733 (735)	410		
*rpoC1*	LSC	432	757	1635 (1611)		
*ycf3*	LSC	124	730 (729)	230	761 (762)	153
*trnL-UAA*	LSC	35	501 (495)	50		
*trnV-UAC*	LSC	39	597	37		
rps12 *	LSC	114	-	232	-	26
*clpP*	LSC	71	833 (830)	292	651 (661)	246
*petB*	LSC	6	801	642		
*petD*	LSC	8	704	496		
*rpl16*	LSC	9	1045 (1054)	399		
*rpl2*	IR	391	667	434		
*ndhB*	IR	777	702	756		
*trnI-GAU*	IR	42	937	35		
*trnA-UGC*	IR	38	802	35		
*ndhA*	SSC	553	1006 (1004)	539		

* *rps12* gene is a trans-spliced gene. Gene length in *A. longecassidatum* Nakai is shown in parentheses. CP: chloroplast; LSC: large single copy; IR: inverted repeat; SSC: small single copy.

**Table 4:** Polymorphic SSRs between the *A. pseudolaeve* Nakai and *A. longecassidatum* Nakai CP genomes. CP: chloroplast; IGS: intergenic sequence.

No.	Location	Region	Motif	Repeat Number	
				*A. pseudolaeve*	*A. longecassidatum*
1	*trnH-psbA*	IGS	A	9	8
2	*trnK-matK*	IGS	T	13	12
3	*trnK-trnQ*	IGS	A	12	10
4	*trnK-trnQ*	IGS	T	9	10
5	*trnG*	intron	T	15	18
6	*atpF*	intron	T	16	14
7	*psbM-trnD*	IGS	T	16	11
8	*psbM-trnD*	IGS	AT	18	12
9	*trnS-psbZ*	IGS	A	11	10
10	*trnG-trnfM*	IGS	A	18	19
11	*psaA-ycf3*	IGS	T	9	10
12	*ycf3*	intron	T	11	12
13	*ycf3*	intron	A	9	8
14	*rps4-trnT*	IGS	A	12	11
15	*trnF-ndhJ*	IGS	T	8	10
16	*ndhC-trnV*	IGS	A	9	11
17	*ndhC-trnV*	IGS	A	14	9
18	*accD-psaI*	IGS	A	9	8
19	*accD-psaI*	IGS	A	13	12
20	*psaI-ycf4*	IGS	T	10	11
21	*ycf4-cemA*	IGS	T	10	9
22	*petA-psbJ*	IGS	T	10	9
23	*psbE-petL*	IGS	T	9	8
24	*psbE-petL*	IGS	A	9	8
25	*psbE-petL*	IGS	T	11	10
26	*rps12-clpP*	IGS	ATT	12	9
27	*clpP*	intron	A	13	11
28	*clpP*	intron	A	14	11
29	*rpl16*	intron	A	12	15
30	*rpl16-rps3*	IGS	T	11	14
31	*ndhF-trnL*	IGS	A	12	11
32	*ndhF-trnL*	IGS	T	12	11
33	*ccsA-ndhD*	IGS	T	13	10
34	*ndhD-psaC*	IGS	A	8	9
35	*ndhG*	Exon	T	11	10
36	*ndhA*	intron	A	11	9

**Table 5:** Distribution of palindromic repeats in the CP genomes of *A. pseudolaeve* Nakai and *A. longecassidatum* Nakai.

Species	Position	Repeat Unit Length (bp)	Repeat Units Sequences	Region
*A. pseudolaeve*	IGS (*trnE-trnT*)	31	TCTATTTCTTATTTCTATATATTCTAATGAT	LSC
	IGS (*petA-psbJ*)	33	GTAAGAATAAGAACTCAATGGACCTTGCCCCTC	LSC
	IGS (*psbT-psbN*)	28	TTGAAGTAAAGTAATGAGCCTCCCATAT	LSC
	IGS (*petD-rpoA*)	24	ATGTATCTAGGGACTAGTCCCTTC	LSC
	Exon (*ycf2*)	24	AGATCCATTAGATAATGAACTATT	IR
	Exon (*ycf15*)	21	TGGTTGTTCGCCGTTCAAGAA	IR
*A. longecassidatum*	IGS (*trnE-trnT*)	31	TCTATTTCTTATTTCTATATATTCTAATGAT	LSC
	IGS (*petA-psbJ*)	33	GTAAGAATAAGAACTCAATGGACCTTGCCCCTC	LSC
	IGS (*psbT-psbN*)	28	TTGAAGTAAAGTAATGAGCCTCCCATAT	LSC
	IGS (*petD-rpoA*)	24	ATGTATCTAGGGACTAGTCCCTTC	LSC
	Exon (*ycf2*)	24	AGATCCATTAGATAATGAACTATT	IR
	Exon (*ycf15*)	21	TGGTTGTTCGCCGTTCAAGAA	IR

CP: chloroplast; IGS: intergenic sequence; LSC: large single copy; IR: inverted repeat region.

**Table 6:** Primer information for insertion and selection (indel) marker development in this study.

Primer Name	Primer Sequence (5′ > 3′)	Position
AcoTT-F	TGC TTA CGA AGT TGT TCC GGC T	*trnK-trnQ*
AcoTT-R	CAC AAA CCA AAT CCG AGT ACC GA	
AcoYN-F	GAT GGA ATC GTC CAT CGC GT	*ycf1-ndhF*
AcoYN-R	TGT AAG TGG AGG ACG GAT CTC T	

## Supplementary Materials

Supplementary Materials are available online.

## Abbreviations

CP	Chloroplast
LSC	Large single copy
SSC	Small single copy
IR	Inverted repeat
tRNA	Transfer RNA
rRNA	Ribosomal RNA
KIOM	Korea Institute of Oriental Medicine
SSRs	Simple Sequence Repeats
SNPs	Single-Nucleotide Polymorphisms
Indel	Insertion and Deletion
