# Supplementary material for: The Complete Chloroplast Genome Sequences of Aconitum pseudolaeve and Aconitum longecassidatum, and Development of Molecular Markers for Distinguishing Species in the Aconitum Subgenus Lycoctonum
Source: Molecules. 2017 Nov 21;22(11):2012. doi: 10.3390/molecules22112012 (PMC6150344; doi:10.3390/molecules22112012)
Supplement: Supplementary file 1 [file molecules-22-02012-s001.pdf]

molecules

Supplementary Materials:

The Complete Chloroplast Genome Sequences of *Aconitum pseudolaeve* and *Aconitum longecassidatum* and development of molecular markers for distinguish species in *Aconitum* Subgenus *Lycoctonum*

Inkyu Park, Sungyu Yang, Goya Choi, Wook Jin Kim, and Byeong Cheol Moon*

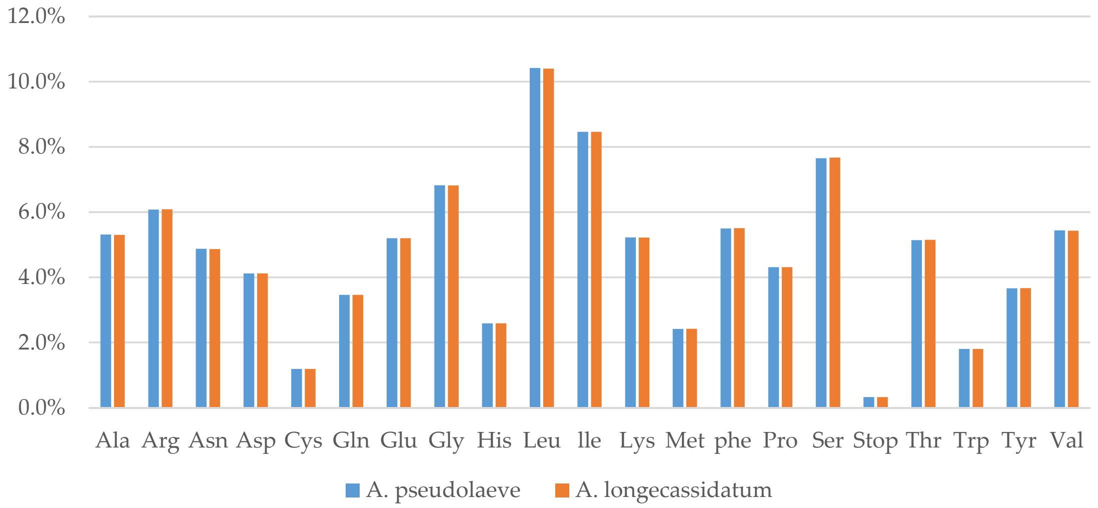

Figure S1 Amino acid frequencies in *A. pseudolaeve* and *A. longecassidatum* protein-coding sequences

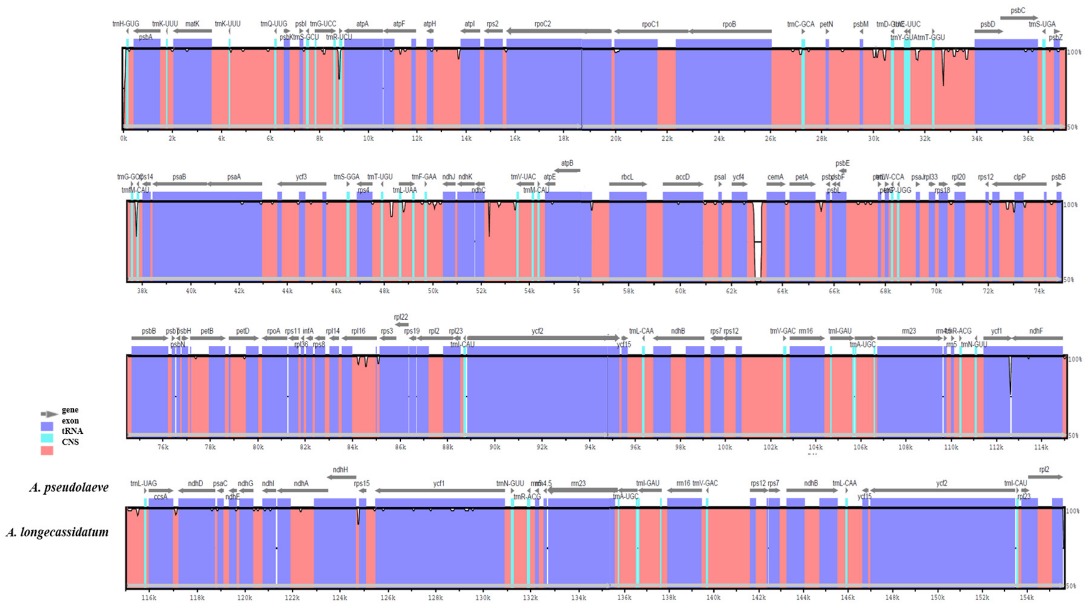

Figure S2 Comparison of *A. pseudolaeve*, *A. longecassidatum* chloroplast genomes using mVISTA. Complete CP genomes of *A. longecassidatum* were compared with *A. pseudolaeve* as reference. Blue block, conserved gene; Sky-blue block, tRNA and rRNA; Red block, intergenic region. White peaks were sequence variation region between *A. pseudolaeve* and *A. longecassidatum*.

Table S1 Assembly information of *A. pseudolaeve* and *A. longecassidatum* CP genomes

Scientific name	Aligned reads (#)	Coverage (x)	Genome length (bp)	# of contig
Aconitum pseudolaeve	405,740	345.6	155,628	7
Aconitum longecassidatum	264,874	222.7	155,524	6

Table S2 Base composition of two *Aconitum* CP genomes

A. pseudolaeve	**A (%)**	**T (%)**	**G (%)**	**C (%)**	**length (bp)**
LSC	31.4	32.5	17.7	18.4	86,683
SSC	34.0	33.4	15.2	17.4	17,091
IR	28.4	28.4	21.6	21.6	51,854
total	30.7	31.2	18.7	19.3	155,628
CDS	30.5	31.2	20.4	17.9	79,377
First position	30.6	23.6	26.9	18.9	26,459
Second position	29.4	32.2	17.9	20.5	26,459
Third position	31.6	37.7	16.5	14.2	26,459
A. longecassidatum					
LSC	31.4	32.5	17.7	18.4	86,466
SSC	34.0	33.3	15.2	17.5	16,950
IR	28.5	28.5	21.5	21.5	52,108
total	30.7	31.2	18.7	19.3	155,524
CDS	30.5	31.2	20.4	17.9	79,461
First position	30.6	23.6	26.9	18.9	26,487
Second position	29.4	32.2	17.9	20.5	26,487
Third position	31.6	37.7	16.5	14.2	26,487

Table S3 Codon-anticodon recognition pattern and codon usage for two *Aconitum* genomes

Amino acid	Codon	*A. pseudolaeve*		*A. longecassidatum*		tRNA
		Count	RSCU*	Count	RSCU	
Phe	UUU	921	1.27	926	1.27	
Phe	UUC	534	0.73	534	0.73	trnF-GAA
Leu	UUA	828	1.8	830	1.81	trnL-UAA
Leu	UUG	586	1.28	586	1.28	trnL-CAA
Leu	CUU	563	1.23	564	1.23	
Leu	CUC	203	0.44	203	0.44	
Leu	CUA	383	0.83	378	0.82	trnL-UAG
Leu	CUG	194	0.42	193	0.42	
Ile	AUU	1083	1.45	1082	1.45	
Ile	AUC	456	0.61	457	0.61	trnI-GAU
Ile	AUA	701	0.94	702	0.94	trnI-CAU<br>trn(f)M-CAU<br>trnM-CAU
Met	AUG	640	1	641	1	trnM-CAU
Val	GUU	526	1.46	526	1.46	
Val	GUC	162	0.45	163	0.45	trnV-GAC
Val	GUA	546	1.52	543	1.51	trnV-UAC
Val	GUG	205	0.57	207	0.58	
Ser	UCU	561	1.66	560	1.65	
Ser	UCC	347	1.03	348	1.03	trnS-GGA
Ser	UCA	413	1.22	414	1.22	trnS-UGA
Ser	UCG	198	0.59	200	0.59	
Pro	CCU	432	1.52	431	1.51	
Pro	CCC	221	0.78	221	0.77	trnS-GCU
Pro	CCA	338	1.19	340	1.19	
Pro	CCG	149	0.52	149	0.52	
Thr	ACU	538	1.58	539	1.58	trnP-UGG
Thr	ACC	252	0.74	253	0.74	
Thr	ACA	418	1.23	420	1.23	
Thr	ACG	152	0.45	152	0.45	trnT-GGU
Ala	GCU	599	1.71	601	1.71	trnT-UGU
Ala	GCC	232	0.66	231	0.66	
Ala	GCA	400	1.14	400	1.14	
Ala	GCG	174	0.5	173	0.49	
Tyr	UAU	768	1.59	771	1.59	trnA-UGC
Tyr	UAC	201	0.41	201	0.41	
Stop	UAA	38	1.33	39	1.36	
Stop	UAG	25	0.87	25	0.87	trnY-GUA

His	CAU	517	1.51	517	1.51	
His	CAC	168	0.49	168	0.49	
Gln	CAA	689	1.51	691	1.51	
Gln	CAG	226	0.49	226	0.49	
Asn	AAU	989	1.53	989	1.54	trnH-GUG
Asn	AAC	300	0.47	299	0.46	trnQ-UUG
Lys	AAA	1004	1.45	1005	1.45	
Lys	AAG	378	0.55	378	0.55	
Asp	GAU	876	1.61	877	1.61	trnN-GUU
Asp	GAC	214	0.39	215	0.39	trnK-UUU
Glu	GAA	1006	1.46	1008	1.46	
Glu	GAG	370	0.54	369	0.54	
Cys	UGU	236	1.5	236	1.5	trnD-GUC
Cys	UGC	79	0.5	79	0.5	trnE-UUC
Stop	UGA	23	0.8	22	0.77	
Trp	UGG	477	1	477	1	
Arg	CGU	364	1.36	364	1.35	trnC-GCA
Arg	CGC	89	0.33	89	0.33	trnW-CCA
Arg	CGA	356	1.33	358	1.33	trnR-ACG
Arg	CGG	120	0.45	120	0.45	
Ser	AGU	391	1.16	395	1.17	
Ser	AGC	115	0.34	115	0.34	
Arg	AGA	500	1.87	502	1.87	trnR-UCU
Arg	AGG	179	0.67	179	0.67	
Gly	GGU	609	1.35	609	1.35	
Gly	GGC	181	0.4	180	0.4	trnG-GCC
Gly	GGA	721	1.6	722	1.6	trnG-UCC
Gly	GGG	295	0.65	295	0.65	

*RSCU – Relative synonymous codon usage

Table S4 Distribution of tandem repeats in two *Aconitum* CP genomes

A. pseudolaeve	**Position**	**Repeat unit length (bp)**	**Repeat unit sequecne**	**Repeat numbers**	**Region**
1	IGS (*rpl2, trnH-GUG*)	17	AATAAATAGAAATAATA	2	LSC
2	IGS (*rpl2, trnH-GUG*)	13	AAATAATAAATAG	2	LSC
3	IGS (*trnK-UUU, rps16*)	17	ACTAATAGTATATATAG	3	LSC
4	IGS (*trnK-UUU, rps16*)	15	TAATAGTATATATAG	3	LSC
5	IGS (*trnS-GCU, trnG-GCC*)	19	TACACATGAAGTCAAGAAA	2	LSC
6	IGS (*trnG-GCC, trnR-UCU*)	18	ATAGGAAAGGCAAAAAAT	2	LSC
7	IGS (*atpF, atpH*)	20	GTTATTGTAGGAGTGAAATC	2	LSC
8	IGS (*rpoB, trnC-GCA*)	21	TGGATCGATCTAGTAATGATG	2	LSC
9	IGS (*psbM, trnD-GUC*)	2	ATATATATATATATATATATATATAT	14	LSC
10	IGS (*trnE-UUC, trnT-GGU*)	22	ATATAGTATAGAAAATAATAAA	2	LSC
11	IGS (*trnT-GGU, psbD*)	15	ATAGTCATTATAATG	2	LSC
12	IGS (*trnT-UGU, trnL-UAA*)	16	TAACATAAATAATATA	2	LSC
13	IGS (*ndhC, trnV-UAC*)	21	CTATTTATTATTTCTATTATA	2	LSC
14	IGS (*accD, psaI*)	21	TTATAGTAATAAGTAATCAAA	2	LSC
15	IGS (*rpl36, rpl36*)	24	ACGTCCATTCTTACGTGAGCCAAT	2	LSC
16	CDS (*ycf2*)	15	TATTGATGATATCAA	3	IR
17	CDS (*ycf2*)	18	ATATTGATGATAGTGACG	3	IR
18	CDS (*ycf2*)	21	AGATAATGAACTATTCAAAGA	2	IR
19	CDS (*ndhG*)	15	TATTACCTATTATAT	2	SSC
20	CDS (*ndhI*)	27	GCTACTTTAAATCAAAACTAAATCTTA	2	SSC
21	CDS (*ycf1*)	21	ATCTATATCTGCTGGTAAATA	2	SSC
A. longecassidatum					
1	IGS (*trnK-UUU,rps16*)	17	ACTAATAGTATATATAG	3	LSC
2	IGS (*trnK-UUU,rps16*)	15	TAATAGTATATATAG	3	LSC
3	IGS (*trnS-GCU,trnG-GCC*)	19	TACACATGAAGTCAAGAAA	2	LSC
4	IGS (*atpF,atpH*)	20	GTTATTGTAGGAGTGAAATC	2	LSC
5	IGS (*rpoB,trnC-GCA*)	21	TGGATCGATCTAGTAATGATG	2	LSC
6	IGS (*trnE-UUC,trnT-GGU*)	22	AGTATAGAAAATAATAAAATAG	2	LSC
7	IGS (*trnT-GGU,psbD*)	15	ATAGTCATTATAATG	2	LSC
8	IGS (*trnT-GGU,psbD*)	22	AAATATTCCTACTTATTAAATGT	2	LSC
9	IGS (*trnG-UCC,trnfM-CAU*)	21	TTAACAGAGTCAGACCTAAAA	2	LSC
10	IGS (*trnT-UGU,trnL-UAA*)	16	TAACATAAATAATATA	2	LSC
11	IGS (*ndhC,trnV-UAC*)	21	CTATTTATTATTTCTATTATA	5	LSC
12	IGS (*accD,psaI*)	21	TTATAGTAATAAGTAATCAAA	2	LSC
13	IGS (*rps11,rps11*)	24	ACGTCCATTCTTACGTGAGCCAAT	2	LSC
14	CDS (*ycf2*)	15	TATTGATGATATCAA	3	IR

15	CDS (*ycf2*)	18	ATATTGATGATAGTGACG	3	IR
16	CDS (*ycf2*)	21	AGATAATGAACTATTCAAAGA	2	IR
17	IGS (*ndhE*,*ndhG*)	15	TATTACCTATTATAT	2	SSC
18	IGS (*ndhG*,*ndhI*)	27	GCTACTTTAAATCAAAACTAAATCTTA	2	SSC
19	CDS (*ycf1*)	21	ATCTATATCTGCTGGTAAATA	2	SSC

Table S5 Indel mutation events between the *A. pseudolaeve* and *A. longecassidatum* CP genomes

No	Location	region	motif	Size	Direction *
1	- *trnH-GUG* (IGS)	LSC	AAATAATAAATAGAAAT	17	Deletion
2	*trnH-GUG - psbA* (IGS)	LSC	A	1	Deletion
3	*trnK-UUU - matK* (IGS)	LSC	T	1	Deletion
4	*trnK-UUU - trnQ-UUG* (IGS)	LSC	AA	2	Deletion
5	*trnK-UUU - trnQ-UUG* (IGS)	LSC	T	2	Insertion
6	*trnG-UCC - trnG-UCC* (intron)	LSC	T	2	Insertion
7	*trnG-UCC - trnG-UCC* (intron)	LSC	TTT	3	Insertion
8	*trnG-UCC - trnR-UCU* (IGS)	LSC	ATAGGAAAGGCAAAAAAT	18	Deletion
9	*atpF - atpF* (intron)	LSC	TTG	3	Insertion
10	*atpF - atpF* (intron)	LSC	A	1	Deletion
11	*atpH - atpI* (IGS)	LSC	ATATAT	6	Insertion
12	*rps2 - rpoC2* (IGS)	LSC	T	1	Insertion
13	*rpoC1* (exon)	LSC	CT	2	Deletion
14	*rpoB - trnC-GCA* (IGS)	LSC	AAA	3	Insertion
15	*psbM - trnD-GUC* (IGS)	LSC	TTTTT	5	Insertion
16	*psbM - trnD-GUC* (IGS)	LSC	TTTTA	5	Deletion
17	*psbM - trnD-GUC* (IGS)	LSC	ATATAT	6	Deletion
18	*trnE-UUC - trnT-GGU* (IGS)	LSC	GATATA	6	Deletion
19	*trnT-GGU - psbD* (IGS)	LSC	TTAAATGTAAATATCCTACTTC	22	Insertion
20	*trnT-GGU - psbD* (IGS)	LSC	TTTT	4	Deletion
21	*trnT-GGU - psbD* (IGS)	LSC	A	1	Deletion
22	*trnT-GGU - psbD* (IGS)	LSC	TTAGTT	6	Deletion
23	*trnS-UGA - psbZ* (IGS)	LSC	A	1	Deletion
24	*trnfM-CAU - rps14* (IGS)	LSC	AAAATTAACAGAGTCAGACCTA	22	Insertion
25	*psaA - ycf3* (IGS)	LSC	T	1	Insertion
26	*ycf3* (exon)	LSC	T	1	Insertion
27	*ycf3 - ycf3* (intron)	LSC	A	1	Deletion
28	*ycf3 - trnS-GGA* (IGS)	LSC	C	1	Insertion
29	*rps4 - trnT-UGU* (IGS)	LSC	A	1	Deletion
30	*trnT-UGU - trnL-UAA* (IGS)	LSC	AAAAGGGTG	9	Deletion
31	*trnL-UAA - trnL-UAA* (intron)	LSC	TACAAA	6	Deletion
32	*trnF-GAA - ndhJ* (IGS)	LSC	TT	2	Insertion
33	*ndhC - trnV-UAC* (IGS)	LSC	TATTATTTCTATTATACTATTTATTATTTCTAT TATACTATTTATTATTTCTATTATACTATT	63	Insertion
34	*ndhC - trnV-UAC* (IGS)	LSC	A	1	Insertion
35	*ndhC - trnV-UAC* (IGS)	LSC	AA	2	Insertion
36	*trnV-UAC - trnV-UAC* (intron)	LSC	AAAAA	5	Deletion
37	*atpB – rbcL* (IGS)	LSC	TAAAG	2	Deletion

38	*accD – psaI* (IGS)	LSC	A	1	Deletion
39	*accD - psaI* (IGS)	LSC	A	1	Deletion
40	*psaI - ycf4* (IGS)	LSC	T	1	Insertion
41	*ycf4 - cemA* (IGS)	LSC	TAAAG	1	Deletion
42	*ycf4 - cemA* (IGS)	LSC	ATCTTGTAATTAAAATTTTTTTTTTTTTATGTT<br>TTGTTTATTTTTATCCTTTCTTTTCCTTTTTGAT<br>GATCAAAAGATTGGATCGTTTATAACTATAA<br>TCATTCTATTTATCTCTTTTTTTGCTACTCGTT<br>TTTGATTTCATCTTATCAATACAAGATTTTCC<br>TAAATTTCCCTCAACTATTCCCCGGCTACGG<br>CTAGCCAGCGAAGTTTTTCGAAATAATTGAT<br>CTAAGGGGGTTCTTTTGCCCCGAAATCAAAA	256	Deletion
43	*petA - psbJ* (IGS)	LSC	TTT	3	Deletion
44	*petA - psbJ* (IGS)	LSC	TTT	3	Deletion
45	*psbE - petL* (IGS)	LSC	A	1	Deletion
46	*psbE - petL* (IGS)	LSC	T	1	Deletion
47	*clpP - clpP* (intron)	LSC	AAAT	4	Insertion
48	*clpP* (exon)	LSC	TATTCG	6	Insertion
49	*clpP - clpP* (intron)	LSC	AAA	3	Deletion
50	*clpP - clpP* (intron)	LSC	AAA	3	Insertion
51	*clpP - clpP* (intron)	LSC	AAATCA	6	Insertion
52	*rps3* (exon)	LSC	TTT	3	Insertion
53	*ycf1 – ndhF* (IGS)	SSC	TAAGAAATGAAATGTCACAATATTTTTTTTAT<br>ACATGTCGAAGTGATGGAAAACAAAAAATC<br>TCTTTTACATATCCACCAAGTTTGTCAACTTT<br>TTTTGAAATGATACAAC	111	Insertion
54	ndhF - *trnL-UAG* (IGS)	SSC	A	1	Deletion
55	*ndhF- trnL-UAG* (IGS)	SSC	A	1	Deletion
56	*ndhF - trnL-UAG* (IGS)	SSC	T	1	Deletion
57	*ndhD* (exon)	SSC	TTT	3	Deletion
58	*psaC* (exon)	SSC	A	1	Insertion
59	*ndhI* (exon)	SSC	T	1	Deletion
60	*ndhA - ndhA* (intron)	SSC	AA	2	Deletion
61	*rps15* (exon)	SSC	ATAATATAAA	10	Insertion

Table S6. SNP mutation events between *A. pseudolaeve* and *A. longecassidatum* CP genomes

No.	Location	Region	*A. pseudolaeve*	*A. longecassidatum*
1	*trnH-GUG* (IGS)	LSC	T	A
2	*trnH-GUG* (IGS)	LSC	A	T
3	*matK* (exon)	LSC	C	T
4	*psbI - trnS-GCU* (IGS)	LSC	A	C
5	*trnG-UCC - trnG-UCC* (intron)	LSC	G	T
6	*trnG-UCC - trnG-UCC* (intron)	LSC	A	C
7	*atpF* (exon)	LSC	T	G
8	*atpH* (exon)	LSC	G	A
9	*atpH - atpI* (IGS)	LSC	G	A
10	*rps2 - rpoC2* (IGS)	LSC	C	A
11	*rpoC1 - rpoC1* (intron)	LSC	A	C
12	*rpoB - trnC-GCA* (IGS)	LSC	C	T
13	*rpoB - trnC-GCA* (IGS)	LSC	A	G
14	*trnC-GCA - petN* (IGS)	LSC	C	T
15	*psbM - trnD-GUC* (IGS)	LSC	A	C
16	*trnE-UUC - trnT-GGU* (IGS)	LSC	T	G
17	*trnT-GGU - psbD* (IGS)	LSC	T	G
18	*trnT-GGU - psbD* (IGS)	LSC	A	C
19	*trnT-GGU - psbD* (IGS)	LSC	T	C
20	*psbC* (exon)	LSC	C	T
21	*psbC* (exon)	LSC	T	C
22	*psbZ* (exon)	LSC	G	T
23	*psaA* (exon)	LSC	C	A
24	*psaA* (exon)	LSC	A	G
25	*trnL-UAA - trnF-GAA* (IGS)	LSC	C	T
26	*trnF-GAA - ndhJ* (IGS)	LSC	C	T
27	*trnF-GAA - ndhJ* (IGS)	LSC	T	C
28	*trnF-GAA - ndhJ* (IGS)	LSC	C	G
29	*trnF-GAA - ndhJ* (IGS)	LSC	T	G
30	*atpE* (exon)	LSC	G	A
31	*accD* (exon)	LSC	C	A
32	*ycf4* (exon)	LSC	T	C
33	*cemA - petA* (IGS)	LSC	T	G
34	*psbE - petL* (IGS)	LSC	T	G
35	*rps18 - rpl20* (IGS)	LSC	A	C
36	*rps12 - clpP* (IGS)	LSC	A	C
37	*clpP - psbB* (IGS)	LSC	G	A
38	*petB - petB* (intron)	LSC	C	G

39	*petD* - *petD* (intron)	LSC	T	C
40	*rpl16* - *rpl16* (intron)	LSC	T	G
41	*rpl16* - *rpl16* (intron)	LSC	G	T
42	*rpl16* - *rps3* (IGS)	LSC	G	A
43	*ndhF* (exon)	SSC	T	C
44	*ndhF* - *trnL-UAG* (IGS)	SSC	T	A
45	*ndhF* - *trnL-UAG* (IGS)	SSC	T	C
46	*ndhF* - *trnL-UAG* (IGS)	SSC	A	C
47	*trnL-UAG* (exon)	SSC	T	G
48	*ndhD* (exon)	SSC	T	G
49	*psaC* (exon)	SSC	A	T
50	*ndhG* (exon)	SSC	T	C
51	*ndhG* - *ndhI* (IGS)	SSC	A	G
52	*ndhI* (exon)	SSC	G	A
53	*ndhI* - *ndhA* (IGS)	SSC	G	A
54	*ycf1* (exon)	SSC	A	C
55	*ycf1* (exon)	SSC	A	C
56	*ycf1* (exon)	SSC	T	C
57	*ycf1* (exon)	SSC	A	G
58	*ycf1* (exon)	SSC	G	T
59	*ycf1* (exon)	SSC	C	A
60	*ycf1* (exon)	SSC	T	C
61	*ycf1* (exon)	SSC	A	T
62	*ycf1* (exon)	SSC	A	C
